# Impact of Masticatory Behaviors Measured With Wearable Device on Metabolic Syndrome: Cross-sectional Study

**DOI:** 10.2196/30789

**Published:** 2022-03-24

**Authors:** Fumiko Uehara, Kazuhiro Hori, Yoko Hasegawa, Shogo Yoshimura, Shoko Hori, Mari Kitamura, Kohei Akazawa, Takahiro Ono

**Affiliations:** 1 Division of Comprehensive Prosthodontics Faculty of Dentistry and Graduate School of Medical and Dental Sciences Niigata University Niigata Japan; 2 School of Food Sciences and Nutrition Mukogawa Women’s University Nishinomiya Japan; 3 Department of Medical Informatics Niigata University Medical and Dental Hospital Niigata Japan

**Keywords:** metabolic syndrome, mastication behaviors, wearable device, daily meal, energy intake, chew, internet of things

## Abstract

**Background:**

It has been widely recognized that mastication behaviors are related to the health of the whole body and to lifestyle-related diseases. However, many studies were based on subjective questionnaires or were limited to small-scale research in the laboratory due to the lack of a device for measuring mastication behaviors during the daily meal objectively. Recently, a small wearable masticatory counter device, called bitescan (Sharp Co), for measuring masticatory behavior was developed. This wearable device is designed to assess objective masticatory behavior by being worn on the ear in daily life.

**Objective:**

This study aimed to investigate the relation between mastication behaviors in the laboratory and in daily meals and to clarify the difference in mastication behaviors between those with metabolic syndrome (MetS) and those without (non-MetS) measured using a wearable device.

**Methods:**

A total of 99 healthy volunteers (50 men and 49 women, mean age 36.4 [SD 11.7] years) participated in this study. The mastication behaviors (ie, number of chews and bites, number of chews per bite, and chewing rate) were measured using a wearable ear-hung device. Mastication behaviors while eating a rice ball (100 g) in the laboratory and during usual meals for an entire day were monitored, and the daily energy intake was calculated. Participants’ abdominal circumference, fasting glucose concentration, blood pressure, and serum lipids were also measured. Mastication behaviors in the laboratory and during meals for 1 entire day were compared. The participants were divided into 2 groups using the Japanese criteria for MetS (positive/negative for MetS or each MetS component), and mastication behaviors were compared.

**Results:**

Mastication behaviors in the laboratory and during daily meals were significantly correlated (number of chews *r*=0.36; *P*<.001; number of bites *r*=0.49; *P*<.001; number of chews per bite *r*=0.33; *P*=.001; and chewing rate *r*=0.51; *P*<.001). Although a positive correlation was observed between the number of chews during the 1-day meals and energy intake (*r*=0.26, *P*=.009), the number of chews per calorie ingested was negatively correlated with energy intake (*r*=–0.32, *P*=.002). Of the 99 participants, 8 fit the criteria for MetS and 14 for pre-MetS. The number of chews and bites for a rice ball in the pre-MetS(+) group was significantly lower than the pre-MetS(–) group (*P*=.02 and *P*=.04, respectively). Additionally, scores for the positive abdominal circumference and hypertension subgroups were also less than the counterpart groups (*P*=.004 and *P*=.01 for chews, *P*=.006 and *P*=.02 for bites, respectively). The number of chews and bites for an entire day in the hypertension subgroup were significantly lower than in the other groups (*P*=.02 and *P*=.006). Furthermore, the positive abdominal circumference and hypertension subgroups showed lower numbers of chews per calorie ingested for 1-day meals (*P*=.03 and *P*=.02, respectively).

**Conclusions:**

These results suggest a relationship between masticatory behaviors in the laboratory and those during daily meals and that masticatory behaviors are associated with MetS and MetS components.

**Trial Registration:**

University Hospital Medical Information Network Clinical Trials Registry R000034453; https://tinyurl.com/mwzrhrua

## Introduction

Recent studies in various fields have revealed that mastication affects various functions in the body, such as obesity prevention [1,2], immune cell differentiation [3,4], and dementia prevention [5]. Among these health effects of mastication, research attention has focused particularly on obesity [6] and diabetes [7,8]. Furthermore, many studies have reported a relationship between chewing frequency, eating speed, and metabolic syndrome (MetS) [9-13]. In these studies, masticatory behavior was also thought to be associated with energy intake. Indeed, Borvornparadorn et al [14] reported that increased number of chews per bite could reduce energy intake.

The relationship between eating behavior, especially fast eating, and MetS or obesity has long been a focus of attention. Some large-scale epidemiological studies also suggested a link between eating speed and obesity and MetS [1,2]. In particular, studies have shown that slower eating speed can reduce energy intake [10,14-18] and that prolonged chewing of food can reduce meal intake [19]. However, most studies were limited to self-administered questionnaires. It is difficult to exactly recognize our own eating behavior (ie, eating fast or slow and less or more chewing). Additionally, assessing mastication behaviors lacks objectivity because of the lack of devices for measuring mastication behaviors during daily meals.

In previous studies, chewing frequency was assessed using dedicated jaw movement measuring devices [20], muscular activity measuring devices (electromyography) [21], videorecordings [22-24], or wearable chewing counting devices [25-27]. However, most of these devices were not developed past the prototype stage, and participants might not eat naturally with these large devices since the requirement for special devices often hinders the evaluation of masticatory movement. Additionally, masticatory behaviors when eating meals while being videorecorded might be different from those during a usual meal.

A simple and accurate mastication measuring device called bitescan (Sharp Co) [28] was developed. The bitescan is a wearable ear hook–type device that monitors daily mastication behavior without disturbing the wearer. Furthermore, this revolutionary device can accurately monitor mastication behavior, namely the number of chews, number of bites, and chewing rate, making it possible to obtain these measurements in the laboratory as well as at home. We have previously confirmed the validity of this mastication measuring device [28].

The aim of this study was to investigate whether masticatory behavior in laboratory had a relationship to that in daily meals and to clarify the relationship between masticatory behavior and MetS by more objectively and accurately monitoring masticatory behavior using the bitescan. We hypothesized that masticatory behaviors in different environments had a relationship and that the presence or absence of MetS was related to masticatory behaviors.

## Methods

### Participants

The participants were 50 healthy men and 49 healthy women, with a mean age of 36.4 (SD 11.7) years, who volunteered for the study. Participants were recruited for the study using advertisements, flyers, and personal communications. We excluded participants who had dysphagia, dental pain, periodontal problems, temporomandibular joint dysfunction syndrome, and those who were receiving dental treatment or medication for lifestyle-related diseases, such as diabetes, hypertension, and hyperlipidemia. These exclusion criteria were confirmed verbally. We explained the purpose of our study, and those who agreed to participate provided written informed consent, which was documented before they entered the study.

**Figure 1:**
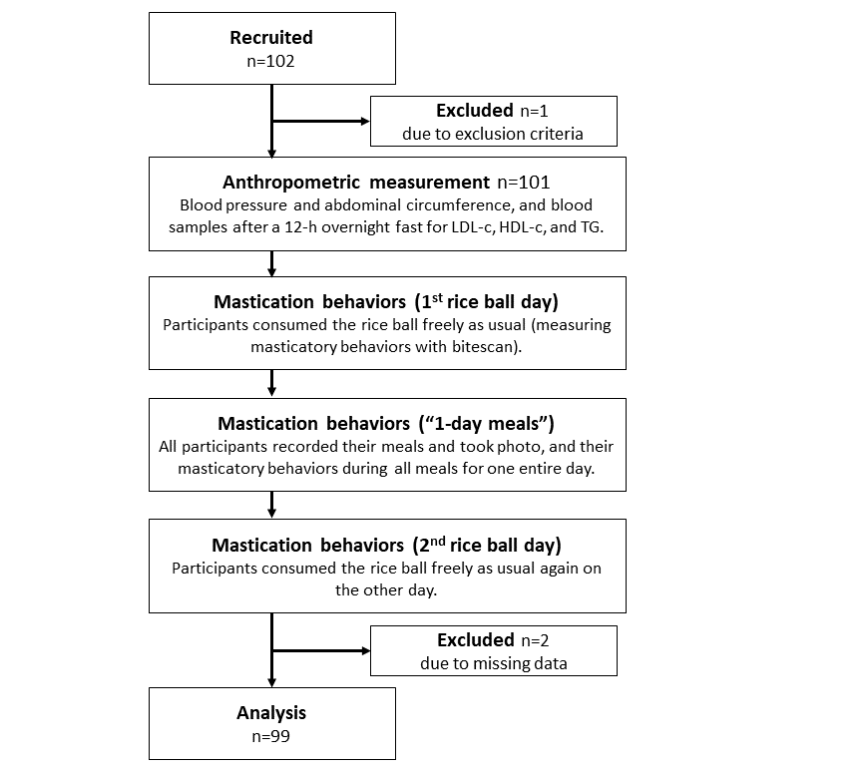
Flow diagram of participant assessment through the trial.

Sample size was estimated based on the number of chews while consuming a rice ball in MetS and non-MetS groups. Results from our preliminarily study (MetS group: mean 167, SD 67; non-MetS group: mean 222, SD 79) provided the effect size as 0.75. The prevalence of metabolic syndrome in Japanese was approximately 20% [29]. Therefore, 94 participants were required for 80% power with a 2-sided 𝛼=.05 for Mann-Whitney *U* test (G*Power 3.1.9.7, Heinrich-Heine-Universität). A total of 102 people applied to this study, 1 was excluded using the exclusion criteria, and 2 people with missing data were excluded; 99 participants were included in the final analysis (Figure 1).

### Ethics Approval

The study conformed to the standards of the Declaration of Helsinki and was approved by the institutional review board of Niigata University (approval number: 2017-0230). The study was registered at the University Hospital Medical Information Network Clinical Trials Registry [R000034453].

### Bitescan Device to Measure Masticatory Behaviors

**Figure 2:**
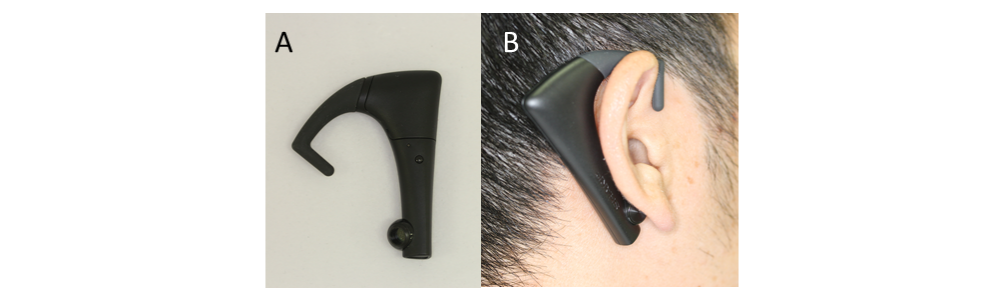
The bitescan: (A) main body and (B) in position.

**Figure 3:**
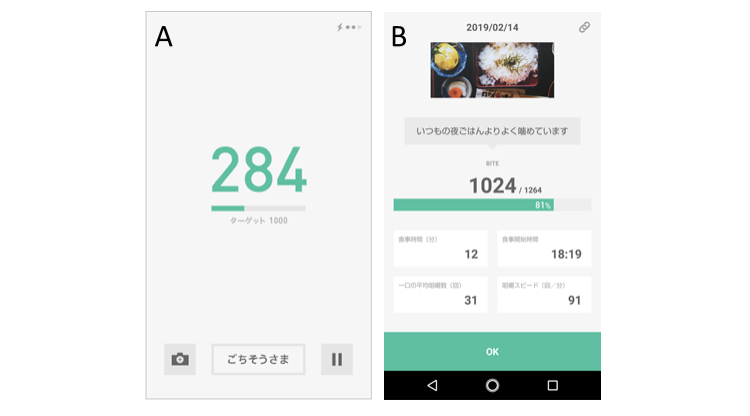
Screenshot of bitescan app: (A) during measurement and (B) showing results of mastication behavior.

The number of chews, number of chews per bite, number of bites, and chewing rate were measured with the bitescan device [28] (Figure 2). This wearable device has an infrared distance sensor and accelerometer and scans the morphological change in the skin surface at 20 Hz on the posterior side of the pinna during mastication. This device is designed to be worn on the right side and has an adjustable ear hook. Three different ear hook sizes (small, medium, and large) were prepared; therefore, we could adjust the device and use the ear hook best suited to each participant’s pinna (Figure 1). Before the measurement, we fit the participants with the bitescan to ensure that the sensor was correctly located on the back of the pinna. The bitescan was then connected to a smartphone (SHM05, Sharp Co) via Bluetooth, and the data were collected with a dedicated smartphone app (Figure 3).

### Data Collection

#### Mastication Behaviors

A rice ball (100 g, Maho Cold Foods Co Ltd) was prepared as the prescribed test food. Each participant was instructed to sit in a chair and relax while the properly sized bitescan was adjusted and we confirmed that it worked properly. Participants were then instructed to consume the rice ball freely, as usual. There were no limitations and no special instructions for participants when consuming the test food regarding the number of chews, chewing rate, or the timing of swallowing; they were asked only to eat the test food as they normally would.

The measurements of the participant chewing the rice ball were taken twice. All participants were also asked to record their masticatory behaviors during all meals for 1 entire day. The 2 rice ball measurements and the 1-day measurement were performed on different days.

#### Energy Intake

All participants completed dietary records accompanied by photographs during the 1-day measurement. Using the photographs, a registered dietitian identified foods and estimated their portion sizes. Energy intake was calculated in accordance with the Standard Tables of Food Composition in Japan 2015 (Seventh Revised Edition) [30].

#### Anthropometric Measurement

The anthropometric measurements were blood pressure (BP) and abdominal circumference (AC), which were measured by the medical staff on the first visit day before measuring masticatory behavior. Blood pressure was measured using a sphygmomanometer (HBP-9020, Omron Corp) with the right arm with participant in the supine position. Abdominal circumference was measured at the midpoint between the iliac crest and rib cage on the midaxillary line using a tape measure. All measurements were performed with participants dressed in light clothing and barefoot.

We also collected blood samples after a 12-hour overnight fast. Fasting glucose concentration (GLU) and serum lipids (SL; low-density lipoprotein cholesterol, high-density lipoprotein cholesterol [HDL-c], and triglycerides) were measured using an automated analyzer.

#### Diagnosis of MetS

We used the Japanese diagnosis of MetS, which is made according to the following criteria [31]:

1. AC: men: ≥0.85 m; women: ≥0.90 m
2. SL: hypertriglyceridemia: ≥1.69 mmol/L or low HDL-c <1.04 mmol/L
3. BP: systolic pressure ≥130 mm Hg or diastolic pressure ≥85 mm Hg
4. GLU: ≥6.1 mmol/L

We divided the participants into the following groups:

- MetS(+) versus MetS(–) groups: criterion 1 and more than two of criteria 2, 3, and 4
- pre-MetS(+) versus pre-MetS(–) groups: criterion 1 and more than one of criteria 2, 3, and 4
- possible MetS(+) versus possible MetS(–) groups: more than one of criteria 1, 2, 3, and 4

Furthermore, in accordance with the above diagnostic criteria for MetS, we also divided participants into the following groups:

- AC(+) versus AC(–) groups according to criterion 1
- SL(+) versus SL(–) groups according to criterion 2
- BP(+) versus BP(–) groups according to criterion 3
- GLU(+) versus GLU(–) groups according to criterion 4

### Statistical Analysis

We analyzed the number of chews, number of bites, number of chews per bite, and chewing rate while participants consumed a rice ball and during the 1-day meals. When recording the data during ingestion of the rice balls, we used the average of the 2 measurements as the representative value. We then calculated the number of chews per calorie ingested for the 1-day measurements.

To compare masticatory behaviors in different environments, the relationship of masticatory behaviors between consuming a rice ball and ingesting all meals for the 1-day measurement was calculated using a Spearman correlation. The masticatory behaviors between the 2 groups were compared using the Mann-Whitney *U* test. Statistical analyses were conducted using SPSS (version 23.0 for Windows, IBM Corp), and the level of significance was set at *P*=.05.

## Results

### Masticatory Behaviors Under Different Conditions

The Spearman correlation coefficients between consuming a rice ball and ingesting the 1-day meals were 0.36 (*P*<.001), 0.49 (*P*<.001), 0.33 (*P*=.001), and 0.51 (*P*<.001) for the number of chews, number of bites, number of chews per bite, and chewing rate, respectively, and significant correlations were observed (Table 1). A positive correlation was observed between the number of chews during the 1-day meals and energy intake (*r*=0.26, *P*=.009). However, the number of chews per calorie ingested was negatively correlated with energy intake for the 1-day meals (*r*=–0.32, *P*=.002). Furthermore, the number of chews per calorie in the 1-day meals showed significant positive correlations with the number of chews, number of chews per bite, number of bites, and the chewing rate while eating a rice ball (*r*=0.48, *P*<.001; *r*=0.20; *P*=.04; *r*=0.32; *P*=.001; and *r*=0.24; *P*=.02, respectively).

The mean number of chews for a rice ball and the 1-day meals were 215 (SD 85) and 2306 (SD 1123), respectively (Table 2 and Multimedia Appendix 1). The number of bites for a rice ball and the 1-day meals were 19.5 (SD 8.0) and 210 (SD 135), respectively (Table 2 and Multimedia Appendix 1). The number of chews per bite (11.7, SD 4.3) and the chewing rate (70.8, SD 7.1) when eating a rice ball were similar to the number of chews per bite (12.4, SD 5.7) and the chewing rate (71.4, SD 7.6) during the 1-day meals.

Female participants had higher numbers of chews (*P*=.009), numbers of bites (*P*<.001) for a rice ball, and numbers of chews per calorie (*P*=.045), a smaller number of chews per bite (*P*=.01 for rice ball, *P*=.02 for 1-day meals), and a slower chewing rate (*P*=.008 for rice ball, *P*=.02 for 1-day meals).

**Table 1:** Relationship between masticatory behaviors in the laboratory and during meals ingested for 1 entire day (n=99).

	Eating a rice ball in the laboratory					Caloric intake for 1 entire day, *r* (*P* value)
	Number of chews, *r* (*P* value)	Number of chews per bite, *r* (*P* value)	Number of bites, *r* (*P* value)	Chewing rate, *r* (*P* value)		
Number of chews	.360 (<.001)	.282 (.005)	.126 (.21)	.328 (.001)	.262 (.009)	
Number of chews per bite	.122 (.23)	.493 (<.001)	–.281 (.005)	.448 (<.001)	.062 (.54)	
Number of bites	.205 (.04)	–.104 (.30)	.334 (.001)	–.038 (.71)	.151 (.14)	
Chewing rate	.185 (.07)	.519 (<.001)	–.222 (.03)	.512 (<.001)	.047 (.65)	
Caloric intake for 1 entire day	–.172 (.09)	.164 (.11)	–.357 (<.001)	.179 (.08)	—^a^	
Number of chews per calorie ingested	.484 (<.001)	.203 (.04)	.324 (.001)	.235 (.02)	–.315 (.002)	

^a^Not applicable.

### Masticatory Behaviors and MetS

Of the participants, 8% (8/99) fulfilled the diagnostic criteria for MetS. There was no significant difference in mastication behaviors between the MetS(+) group and MetS(–) groups.

A total of 14% (14/99) of participants fulfilled the diagnostic criteria for pre-MetS. The numbers of chews (*P*=.02) and bites (*P*=.04) of a rice ball for the pre-MetS(+) group were significantly lower than for those in the pre-MetS(–) group. However, the 1-day mastication behaviors were not significantly different between the pre-MetS(+) and pre-MetS(–) groups.

A total of 53% (52/99) met one or more criteria for a MetS diagnosis. The possible MetS(+) group showed significantly lower numbers of chews and bites for both the rice ball (*P*<.001, *P*=.001) and 1-day meals (*P*=.007, *P*=.008) and a lower number of chews per calorie ingested (*P*=.005) than the possible MetS(–) group.

**Table 2:** Masticatory behavior while eating a rice ball in the laboratory.

			n		Number of chews				Number of chews per bite				Number of bites				Chewing rate (/min)		
					median (IQR)		*P* value		median (IQR)		*P* value		median (IQR)		*P* value		median (IQR)		*P* value
All			99		192 (106)		—^a^		10.7 (5.1)		—		18.0 (7.5)		—		70.7 (8.6)		—
**Sex**			—		—		.009		—		.01		—		<.001		—		.008
	Men	50		172 (106)		—		11.7 (7.6)		—		15.0 (7.5)		—		72.3 (10.2)		—	
	Women	49		208 (106)		—		10.1 (3.6)		—		21.0 (9.0)		—		70.1 (9.3)		—	
**MetS^b^**			—		—		.24		—		.83		—		.39		—		.25
	Yes	8		171 (67)		—		11.6 (7.7)		—		16.5 (5.8)		—		72.9 (12.9)		—	
	No	91		199 (110)		—		10.7 (5.1)		—		18.5 (8.0)		—		70.4 (9.2)		—	
**pre-MetS**			—		—		.02		—		.92		—		.04		—		.39
	Yes	14		162 (56)		—		11.6 (7.2)		—		15.0 (8.5)		—		71.7 (10.7)		—	
	No	85		202 (114)		—		10.7 (5.0)		—		19.0 (9.0)		—		70.4 (8.8)		—	
**possible MetS**			—		—		<.001		—		.74		—		.001		—		.92
	Yes	52		174 (84)		—		10.6 (5.8)		—		16.3 (7.3)		—		71.4 (9.9)		—	
	No	47		214 (114)		—		10.8 (4.6)		—		20.5 (9.5)		—		70.3 (8.6)		—	
**Abdominal circumference (m)**			—		—		.004		—		.90		—		.006		—		.86
	AC^c^(+)	24		166 (80)		—		10.9 (7.1)		—		15.0 (7.5)		—		71.2 (10.5)		—	
	AC(–)	75		206 (118)		—		10.7 (4.8)		—		20.0 (10.5)		—		70.4 (8.5)		—	
**Serum lipid (mmol/L)**			—		—		.04		—		.87		—		.15		—		.73
	SL^d^(+)	13		161 (48)		—		11.7 (7.9)		—		17.0 (9.5)		—		71.3 (14.1)		—	
	SL(–)	86		203 (113)		—		10.7 (4.9)		—		18.5 (8.6)		—		70.5 (8.4)		—	
**Blood pressure (mm Hg)**			—		—		.01		—		.91		—		.02		—		.91
	BP^e^(+)	36		168 (84)		—		10.9 (5.6)		—		16.3 (6.4)		—		71.4 (9.9)		—	
	BP(–)	63		206 (113)		—		10.7 (4.9)		—		20.0 (10.0)		—		70.4 (8.5)		—	
**Fasting glucose (mmol/L)**			—		—		.77		—		.76		—		.65		—		.40
	GLU^f^(+)	7		208 (90)		—		11.5 (5.2)		—		17.0 (11.0)		—		73.4 (6.5)		—	
	GLU(–)	92		191 (110)		—		10.7 (5.2)		—		18.3 (7.9)		—		70.4 (9.4)		—	

^a^Not applicable.

^b^MetS: metabolic syndrome.

^c^AC: abdominal circumference (men ≥0.85 m, women ≥0.90 m).

^d^SL: serum lipids (triglyceride ≥1.69 mmol/L or HDL cholesterol <1.04 mmol/L).

^e^BP: blood pressure (systolic pressure ≥130 mm Hg or diastolic pressure ≥85 mm Hg).

^f^GLU: fasting glucose concentration (≥6.1 mmol/L).

For abdominal circumference, 24% (24/99) of participants exceeded the criteria. The AC(+) group showed significantly lower numbers of chews (*P*=.004) and bites (*P*=.006) when ingesting a rice ball and a lower number of chews per calorie (*P*=.03) ingested than the AC(–) group.

For serum lipids, 13% (13/99) of participants exceeded the criteria. The number of chews of a rice ball in the SL(+) group was significantly lower than in the SL(–) group (*P*=.04).

For blood pressure, 36% (36/99) of participants exceeded the criteria. The BP(+) group showed significantly lower numbers of chews (*P*=.01, *P*=.02) and bites (*P*=.02, *P*=.006) for both a rice ball and 1-day meals and a significantly lower number of chews per calorie (*P*=.02) ingested than the BP(–) group.

For blood glucose concentration, 7% (7/99) of participants exceeded the criteria. There was no significant difference in the masticatory behaviors between the GLU(+) and GLU(–) groups except for the number of bites for the 1-day meals.

## Discussion

### Principal Findings

In this study using a wearable mastication monitoring device, we identified baseline mastication behaviors, which have not been determined previously. To our knowledge, there are no studies investigating the relationship between mastication behaviors eating usual meals and eating in the laboratory. This was also the first time that this many participants were objectively evaluated regarding their eating behavior, including the numbers of chews and bites, and the first time a relationship between masticatory behaviors and MetS has been examined. Therefore, we believe that this study monitoring daily dietary mastication behaviors using wearing device can be considered innovative research.

### Mastication Behaviors and Environment

In most studies evaluating the number of chews, participants consumed only a prescribed meal in the laboratory, which was considered different from usual meals or eating behaviors. Additionally, masticatory behaviors when eating meals while being videorecorded might be different from those during a usual meal.

Although Zhang et al [32] measured mastication behavior in the lab and home environment for assessment of detection of mastication, they did not investigate the relationship. Petty et al [33] found that self-reported eating rate aligned with the eating rate measured in laboratory but not with free-living meals. For these reasons, we compared the masticatory behavior in the laboratory environment with that in the normal environment. We found the number of chews per bite (rice ball: 11.7, SD 4.3; 1-day meals: 12.4, SD 5.7) and chewing rate (rice ball: 70.8, SD 7.1; 1-day meals: 71.4, SD 7.6) had significant correlation and were almost the same. In our study, the contents of daily meals were not regulated, and the participants ingested as usual. The amount and physical characteristics of food affect mastication behavior, and mastication behavior varies greatly among individuals. However, even when comparing the results for each individual, the correlation between the number of chews per bite and the chewing rate between the laboratory environment and the usual daily environment was approximately 0.50, a moderate-intensity correlation.

Accordingly, the masticatory behavior in the laboratory had significant correlation with the participants’ usual masticatory behaviors, suggesting that the chewing behavior in daily life can be inferred from the chewing behavior of rice balls in the laboratory.

We confirmed that our ear hook–type device hangs only on the pinna and has little effect on eating behavior; however, it is difficult to make comparisons of eating behavior with no device. Additionally, participants’ awareness of masticatory behavior monitoring might affect outcomes. To avoid this, long-term measurements should be performed to allow participants to become accustomed to the device. However, similarly, the effects of attaching electrodes to the masseter and temporalis muscles and eating while being videorecorded have not been investigated.

In our study, the mean number of chews per day was 2306 (SD 1123). To our knowledge, few studies have measured and reported the number of chews during usual meals or within 1 entire day. This result is an indicator of the masticatory behaviors of Japanese people eating their daily diet.

The correlation between the number of chews while eating a rice ball and the 1-day meals was 0.36, and the correlation coefficient of the number of bites was 0.33, which indicated a weak correlation. In other words, generally, the number of chews and the number of bites when eating usual meals did not change even when eating a rice ball in the laboratory. However, we should also consider the amount of dietary intake.

As a result of investigating nutritional intake, we found those who chewed more tended to have a large energy intake. In other words, we expected that people who ate a large amount of food in a day would chew a lot. Borvornparadorn et al [14] measured energy intake using regulated food in a laboratory and investigated the relationship between the amount of mastication and calorie intake. However, as far as we know, no studies have measured both the energy intake of daily meals and mastication behaviors. The amount of food was considered to have a great influence on masticatory behavior. However, it was difficult to measure the volume and weight of meals in this study. Petty et al [33] reported eating rate calculated in calories per minute but not volume or weight. Due to differences in the amount of food people eat, we decided to calculate the number of chews per calorie ingested. As a result, a moderate correlation coefficient of 0.48 was obtained for the number of chews of a rice ball. Furthermore, the number of chews per calorie ingested showed a significant correlation with the other items measured when participants ingested rice balls. Therefore, the investigation of the number of chews per calorie intake appears to be meaningful.

In this research, we chose rice balls as the test food after consideration that preference and tableware [34] might affect masticatory behaviors. Rice balls have long been popular as a convenient food for Japanese people, although they may not be a familiar food internationally. We also chose rice balls because these were easily accepted by the Japanese and could be used as a prescribed amount of test food. Asian people generally use chopsticks for eating, but rice balls are usually ingested using the hands.

### Relationship Between MetS and Masticatory Behavior

A significant relationship between MetS and eating speed [1,2,35,36] has been reported previously. However, most of these studies were limited to participant self-reported assessment of eating behavior. Regarding self-reported eating behavior, Woodward et al [37] reported a discrepancy between self-reporting and objective observation on eating rate. Furthermore, masticatory performance was reported to be associated with the prevalence of MetS [38,39]. These previous reports suggested that mastication was associated with MetS through nutrition and feeling full. On the other hand, our results showed no significant difference in the number of chews between the MetS(+) and MetS(–) groups. One of the reasons is that the percentage of participants in our study with MetS (8%) was much lower than the Japanese prevalence of MetS [29]. However, we found a difference in the numbers of chews and bites of a rice ball between the pre-MetS(+) group and pre-MetS(–) group. Furthermore, the possible MetS(+) group showed significantly lower numbers of chews and bites for both the rice ball and 1-day meals and a lower number of chews per calorie ingested than the possible MetS(–) group. MetS arises from a combination of factors that result in obesity, hypertension, serological abnormalities, and abdominal obesity. It is impossible to explain such complicated pathological conditions with one component (chewing), but we suspect that masticatory behavior may have an effect on lifestyle-related diseases.

Abdominal circumference is an indicator associated with obesity and is the most important factor in MetS. One study reported that it was difficult to obtain a feeling of fullness during meals if the number of chews was low or when participants ate faster [14]. As a result, daily energy intake increased significantly, which was thought to cause obesity [40]. Some studies reported that the amount of food and snack intake decreased significantly with an increase in the number of chews and the chewing time [19,41]. In our study, the number of chews while consuming a rice ball was significantly lower in the AC(+) group. In addition, although no significant difference was observed, the number of chews per day was less in the AC(+) group and the daily energy intake was higher, which may support the above consideration. Differences in the number of chews of rice balls and the number of chews per calorie ingested in the 1-day meals suggested that the number of chews per unit amount might be associated with obesity.

In contrast, no significant difference in the number of chews per bite was observed in this study. In Japan, enlightenment activities with an emphasis on the number of chews per bite such as “chew 30 times per bite” have been recommended. However, considering the results of our study, the number of chews per bite may not be directly related to obesity. In addition, from our results, the number of bites in the AC(+) group was significantly lower. This suggested that the AC(+) group ingested large bites (ie, we considered that the number of chews decreased as a result of taking a large amount into the oral cavity and eating it with a modest number of chews). From these findings, the instruction not only to increase the number of chews but also to reduce the amount taken in one bite might be effective. According to a report by Fukuda et al [40], the number of chews per bite did not change even if the amount per bite increased; therefore, if the amount per bite was large, the total mealtime duration would be shorter and the total number of chews would be lower when eating the same amount of food. In addition, some studies reported that a reduction in the size of the bite helped prevent obesity [42,43].

In our study, there was no difference between the GLU(+) group and the GLU(–) group except for the number of bites in the 1-day meals. In our study, the GLU(+) group constituted only 7 participants (7%). However, some reports suggested a relationship between diabetes and mastication. Masticatory movement promotes glucagon-like peptide-1 secretion, which leads to rapid insulin secretion and may also affect dietary sugar absorption [44,45]. In addition, Read et al [46] suggested that the easiest way to avoid raising the blood sugar level even after eating was to swallow without chewing. Although it was impossible to swallow without chewing for all of the usual meals, in the study by Ranawana et al [47], which compared 15 and 30 chews during a rice meal, fewer chews resulted in a lower postprandial total blood and glycemic index. In contrast, Sato et al [48] reported that frequent chews suppressed the rise in postprandial blood glucose concentration and promoted insulin secretion. As described above, there are many conflicting reports regarding mastication and blood glucose concentration. Furthermore, obesity, diabetes, and postprandial blood glucose responses vary greatly depending on race and sex [49]. Because many complex factors might be involved, further research is required.

In our study, blood pressure was the second factor most associated with mastication after obesity. Studies report that slower and more thorough chewing and eating could increase eating-induced heat production, which could increase systemic metabolism [50]. In our study, the BP(+) group showed significantly lower numbers of chews and bites when ingesting rice balls, lower numbers of chews and bites per day, and a lower number of chews per calorie ingested.

A lower number of chews could affect taste perception because the food is not sufficiently crushed and exposure time of the food in the oral cavity is short. Bolhuis et al [51] reported a relationship between saltiness and appetite using two types of soup with different salinity and density. According to the study, longer exposure times to the oral sensation led to lower soup intake. The authors also reported that the exposure time to the oral sensation had a greater effect than salinity. Increasing the exposure to taste buds per food unit might be effective in reducing food intake. Therefore, it is possible that a participant who chewed less and had a short exposure time in the oral cavity regarding the taste stimulus of the food might have ingested more food. One of the major reasons for increased blood pressure is excessive salt intake; therefore, low masticatory behavior might be related to blood pressure.

Diabetes, hyperlipidemia, and hypertension may have multiple forms related to obesity and arteriosclerosis. Furthermore, MetS is related to lifestyle issues, such as diet, exercise, drinking, smoking, and stress. It is known that negative emotions such as anger, fear, and sadness have been associated with increased impulsive eating and consumption of unhealthy foods [52]. In this study, we investigated only masticatory behaviors and dietary energy intake, and multivariate analysis including these factors is needed. Nevertheless, mastication behavior is a lifestyle issue, and it should receive attention not only regarding the amount and kind of food eaten, but also regarding the way of eating. Our results suggested that masticatory behavior when eating a test meal in the laboratory as well as in the participants’ usual daily diet was associated with MetS. This was a cross-sectional study, and a longitudinal study is necessary to examine whether people with poor masticatory behaviors are more likely to develop MetS or whether better masticatory behaviors are useful for preventing MetS. Furthermore, it is necessary to consider whether masticatory behavior can be transformed by certain interventions.

Although this was a cross-sectional study and other MetS-related factors should be considered, our results suggested that mastication behavior might be related to MetS and MetS components. These results help clarify the relationship between masticatory behaviors, metabolic syndrome, and energy intake. We will perform multivariate analysis for each item related to MetS in the future. The wearable chewing counter that we developed was useful for monitoring masticatory behavior in daily meals. In addition, the smartphone app connected to this device can be equipped with a masticatory behavior change algorithm. Masticatory behavior targets selected for each participant and wearable devices that are easy to wear should lower the hurdles for intervention studies and could contribute to the evidence of a relationship between masticatory behavior and health and the effects of masticatory behavior change. Furthermore, the mastication data measured by this wearable device can send the user’s information to medical personnel by using the internet environment. Medical personnel could provide support for improving daily life, such as sending advice based on this information. The usefulness of mastication could be examined using the big data collected in this way. We believe that this device and app could be used in the future as an approach to monitor and change daily masticatory habits.

### Conclusions

In this study, we investigated mastication behaviors (ie, number of chews and bites, number of chews per bite, and chewing rate) measured using a wearable ear-hung device. We found a significant correlation between mastication behaviors in the laboratory and in daily meals, which are different environments. Furthermore, a significant correlation was observed between the number of chews during the 1-day meals and energy intake and between the number of chews per calorie ingested and energy intake. Neither the pre-MetS obesity nor the hypertension group had a lower number of chews, bites, and chews per calorie. These results suggest that masticatory behaviors are related to MetS and MetS components.

## Appendix

Multimedia Appendix 1 Masticatory behavior during meals ingested for one entire day.

## Abbreviations

AC: abdominal circumference
BP: blood pressure
GLU: fasting glucose concentration
HDL-c: high-density lipoprotein cholesterol
MetS: metabolic syndrome
SL: serum lipids
