# Supplementary material for: Impact of Masticatory Behaviors Measured With Wearable Device on Metabolic Syndrome: Cross-sectional Study
Source: JMIR Mhealth Uhealth. 2022 Mar 24;10(3):e30789. doi: 10.2196/30789 (PMC8990367; doi:10.2196/30789)
Supplement: Multimedia Appendix 1 [file mhealth_v10i3e30789_app1.docx]

| Multimedia Appendix 1. Masticatory behavior during meals ingested for one entire day. | | | | | | | | | | | | | | | | | | | | |
| --- | --- | --- | --- | --- | --- | --- | --- | --- | --- | --- | --- | --- | --- | --- | --- | --- | --- | --- | --- | --- |
|  |  |  | Ingesting meals for 1 entire day (1-day meals) | | | | | | | | | | | | | | | | | |
|  |  | n | Number of chews | | | Number of chews per bite | | | Number of bites | | | Chewing rate (/min) | | | Number of chews per calorie ingested | | | Caloric intake within 1 entire day | | |
|  |  |  | median | IQR | *P* | median | IQR | *P* | median | IQR | *P* | median | IQR | *P* | median | IQR | *P* | median | IQR | *P* |
|  |  |  |  |  |  |  |  |  |  |  |  |  |  |  |  |  |  |  |  |  |
| All |  | 99 | 2024 | 1182 |  | 11.5 | 5.3 |  | 172 | 105 |  | 71.0 | 9.3 |  | 1.26 | 0.75 |  | 1751 | 522 |  |
|  |  |  |  |  |  |  |  |  |  |  |  |  |  |  |  |  |  |  |  |  |
| Sex | Men | 50 | 2072 | 1211 | *.524* | 12.4 | 6.2 | *.018* | 170 | 123 | *.155* | 72.8 | 11.7 | *.010* | 1.16 | 0.72 | *.045* | 1929 | 520 | *<.001* |
|  | Women | 49 | 2024 | 1237 |  | 10.5 | 4.6 |  | 187 | 94 |  | 70.4 | 8.2 |  | 1.39 | 0.83 |  | 1564 | 443 |  |
|  |  |  |  |  |  |  |  |  |  |  |  |  |  |  |  |  |  |  |  |  |
| MetS | yes | 8 | 2306 | 993 | *.232* | 9.6 | 4.9 | *.397* | 222 | 155 | *.064* | 68.7 | 10.8 | *.719* | 1.22 | 0.29 | *.898* | 1844 | 760 | *.329* |
|  | no | 91 | 1999 | 1240 |  | 11.7 | 5.2 |  | 170 | 103 |  | 71.1 | 9.2 |  | 1.26 | 0.81 |  | 1751 | 537 |  |
|  |  |  |  |  |  |  |  |  |  |  |  |  |  |  |  |  |  |  |  |  |
| pre-MetS | yes | 14 | 2000 | 943 | *.609* | 11.4 | 5.8 | *.861* | 169 | 113 | *.688* | 71.7 | 11.6 | *.740* | 1.14 | .42 | *.129* | 1879 | 558 | *.213* |
|  | no | 85 | 2024 | 1334 |  | 11.5 | 5.1 |  | 178 | 105 |  | 71.0 | 9.1 |  | 1.34 | .82 |  | 1712 | 549 |  |
|  |  |  |  |  |  |  |  |  |  |  |  |  |  |  |  |  |  |  |  |  |
| possible-MetS | yes | 52 | 1906 | 1002 | *.007* | 11.7 | 5.5 | *.561* | 164 | 109 | *.008* | 71.3 | 10.6 | *.420* | 1.13 | .72 | *.005* | 1796 | 429 | *.594* |
|  | no | 47 | 2473 | 1429 |  | 11.2 | 4.3 |  | 197 | 133 |  | 71.0 | 8.1 |  | 1.43 | .78 |  | 1701 | 635 |  |
|  |  |  |  |  |  |  |  |  |  |  |  |  |  |  |  |  |  |  |  |  |
| Abdominal circumference | AC(+) | 24 | 1885 | 940 | *.193* | 11.4 | 5.6 | *.788* | 175 | 100 | *.636* | 71.0 | 9.8 | *.813* | 1.13 | .64 | *.026* | 1842 | 517 | *.288* |
|  | AC(−) | 75 | 2076 | 1341 |  | 11.5 | 4.8 |  | 172 | 105 |  | 71.0 | 9.5 |  | 1.38 | .89 |  | 1712 | 552 |  |
|  |  |  |  |  |  |  |  |  |  |  |  |  |  |  |  |  |  |  |  |  |
| Serum lipid concentration | SL(+) | 13 | 1965 | 960 | *.889* | 11.7 | 6.4 | *.983* | 178 | 96 | *.698* | 70.1 | 14.6 | *.717* | 1.13 | 0.52 | *.175* | 1884 | 632 | *.120* |
|  | SL(−) | 86 | 2032 | 1301 |  | 11.4 | 5.1 |  | 172 | 107 |  | 71.1 | 9.0 |  | 1.32 | 0.80 |  | 1707 | 547 |  |
|  |  |  |  |  |  |  |  |  |  |  |  |  |  |  |  |  |  |  |  |  |
| Blood pressure | BP(+) | 36 | 1906 | 1100 | *.020* | 11.3 | 5.4 | *.774* | 161 | 93 | *.006* | 70.9 | 10.5 | *.689* | 1.13 | 0.63 | *.021* | 1810 | 458 | *.551* |
|  | BP(−) | 63 | 2151 | 1320 |  | 11.7 | 5.2 |  | 194 | 111 |  | 71.1 | 9.1 |  | 1.39 | 0.86 |  | 1701 | 552 |  |
|  |  |  |  |  |  |  |  |  |  |  |  |  |  |  |  |  |  |  |  |  |
| Fasting glucose concentration | GLU(+) | 7 | 2311 | 1155 | *.110* | 9.6 | 4.2 | *.421* | 241 | 185 | *.037* | 69.4 | 10.8 | *.682* | 1.34 | 0.72 | *.405* | 1871 | 653 | *.478* |
|  | GLU(−) | 92 | 1978 | 1234 |  | 11.6 | 5.3 |  | 171 | 100 |  | 71.1 | 9.3 |  | 1.23 | 0.79 |  | 1737 | 534 |  |
|  |  |  |  |  |  |  |  |  |  |  |  |  |  |  |  |  |  |  |  |  |
| MetS: metabolic syndrome, IQR: interquartile range | | | | | | | | | | | | | | | | | | | | |
| AC: abdominal circumference (men ≥0.85 m, women ≥0.90 m) | | | | | | | | | | | | | | | | | | | | |
| SL: serum lipids (triglyceride ≥1.69 mmol/L and/or HDL cholesterol <1.04 mmol/L) | | | | | | | | | | | | | | | | | | | | |
| BP: blood pressure (systolic pressure ≥130 mmHg and/or diastolic pressure ≥85 mmHg) | | | | | | | | | | | | | | | | | | | | |
| GLU: fasting glucose concentration (≥6.1 mmol/L) | | | | | | | | | | | | | | | | | | | | |
